# Chronic intermittent hypoxia-mediated renal sympathetic nerve activation in hypertension and cardiovascular disease

**DOI:** 10.1038/s41598-018-36159-9

**Published:** 2018-12-18

**Authors:** Keiko Takahashi, Seiji Ueda, Takashi Kobayashi, Akira Nishiyama, Yoshihide Fujisawa, Takeshi Sugaya, Satomi Shiota, Kazuhisa Takahashi, Tomohito Gohda, Satoshi Horikoshi, Yusuke Suzuki

**Affiliations:** 1 Department of Nephrology, Juntendo University Faculty of Medicine, Tokyo, Japan; 2 Department of Pharmacology, Faculty of Medicine, Kagawa University, Kagawa, Japan; 3 Life Science Research Center, Faculty of Medicine, Kagawa University, Kagawa, Japan; 4 Department of Respiratory Medicine, Juntendo University Faculty of Medicine, Tokyo, Japan

## Abstract

In sleep apnea syndrome (SAS), chronic intermittent hypoxia (CIH) is believed to activate the sympathetic nerve system, and is thus involved in cardiovascular diseases (CVD). However, since patients with SAS are often already obese, and have diabetes and/or hypertension (HT), the effects of CIH alone on sympathetic nerve activation and its impacts on CVD are largely unknown. We, therefore, examined the effects of CIH on sympathetic nerve activation in non-obese mice to determine whether renal sympathetic nerve denervation (RD) could ameliorate CIH-mediated cardiovascular effects. Male C57BL/6 (WT) mice were exposed to normal (FiO_2_ 21%) or CIH (10% O_2_, 12 times/h, 8 h/day) conditions for 4 weeks with or without RD treatment. Increased urinary norepinephrine (NE), 8-OHdG, and angiotensinogen levels and elevated serum asymmetric dimethyl arginine levels were observed in the CIH model. Concomitant with these changes, blood pressure levels were significantly elevated by CIH treatment. However, these deleterious effects by CIH were completely blocked by RD treatment. The present study demonstrated that CIH-mediated renal sympathetic nerve activation is involved in increased systemic oxidative stress, endothelial dysfunction, and renin-angiotensin system activation, thereby contributing to the development of HT and CVD, thus could be an important therapeutic target in patients with SAS.

## Introduction

Sleep apnea syndrome (SAS) is a disorder characterized by recurrent arousal from sleep and intermittent hypoxia (IH). It is considered that such chronic intermittent hypoxia (CIH) may cause similar tissue damage in ischemia-reperfusion^1^. Nocturnal IH attributable to SAS activates the sympathetic nerve system^2,3^ and the renin–angiotensin system (RAS)^4,5^, and causes oxidative stress^2,6^. This cycle may promote vascular inflammation and endothelial dysfunction, thus contributing to the development of atherosclerosis^2,7^.

The influences of SAS are not confined to the night. SAS is sustained during the diurnal cycle and is accompanied by chronically elevated sympathetic nerve activity^8^. Patients with SAS exhibit higher chronic sympathetic nerve activation during periods of normal oxygenation, indicating that a part of the adaptive response remains after the end of the hypoxic condition^3^. In addition, some studies have provided evidence that sympathetic activation represents a ringleader of the essential hypertensive state. These studies^8,9^ show that adrenergic neural factors may be involved in the development and progression of the hypertensive state and its complications. In general, SAS is also known to be associated with hypertension (HT)^10^, left ventricular hypertrophy, and cognitive dysfunction^2^. It is thought that heightened sympathetic nerve activity is a major contributor to SAS-induced HT and, ultimately, cardiovascular disease–associated end-organ damage^11^. Indeed, accumulating evidence from clinical and basic research has indicated the effectiveness of renal sympathetic nerve denervation (RD) in lowering blood pressure, reducing left ventricular hypertrophy, and decreasing the RAS activity^12,13^. Moreover, it has also been shown that RD improves glucose metabolism, insulin sensitivity^14^, and resistant HT in patients with SAS in the absence of any changes in body weight or lifestyle^15^. Such lines of evidence strongly suggest that CIH-induced sympathetic nerve activation may enhance RAS and oxidative stress, being involved in the development of vascular injury, and therefore RD could be a novel therapeutic strategy in preventing cardiovascular diseases (CVD) in patients with SAS. However, since patients with SAS are often already obese, and have diabetes and/or HT, the sole effects of CIH on sympathetic nerve activation and its impacts on CVD are not fully understood. To exclude these confounders, we investigated here the involvement of CIH on the activation of the sympathetic nerve in a non-obese CIH mouse model and its impacts on RAS, oxidative stress and asymmetric dimethylarginine (ADMA), a surrogate marker for CVD, by performing RD in this model.

## Results

### Urinary NE in CIH model with or without renal denervation (RD)

**Figure 1:**
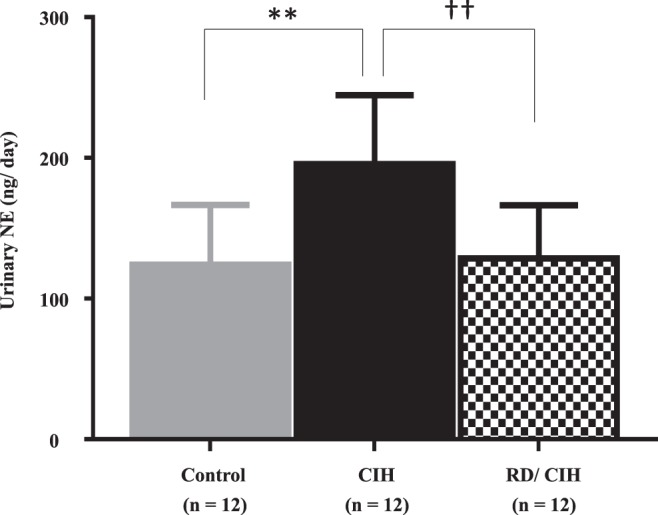
Urinary norepinephrine (NE) levels: Urinary NE levels at week 4 were significantly increased by chronic intermittent hypoxia (CIH) exposure and suppressed by renal denervation (RD) treatment. **P < 0.01 (control vs. CIH). ^††^P < 0.01 (CIH vs. RD/CIH).

Since urinary NE is considered to be an indicator of sympathetic activity, we first evaluated NE concentrations to test the impact of CIH on sympathetic activity. As shown in Fig. 1, the urinary NE levels were significantly increased after CIH exposure, and the NE elevation was abolished by RD.

### Effects of CIH with or without RD on blood pressure

**Figure 2:**
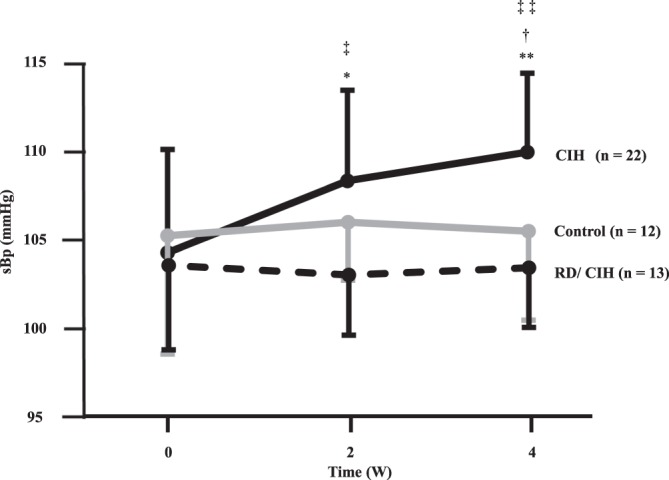
Time-related changes in systolic blood pressure (sBP): Systolic blood pressure levels were increased in CIH model, which were reduced by RD treatment. *P < 0.05 **P < 0.01 (week 0 vs. weeks 2 and 4). ^†^P < 0.05 (control vs. CIH). ^‡^P < 0.05 ^‡‡^P < 0.01 (CIH vs. RD/CIH). sBP: systolic blood pressure, w- weeks.

As shown in Fig. 2, all groups showed the same degree of sBP levels at the beginning of the study. RD did not affect baseline sBP levels (Fig. 2) or body weight and food intake (data not shown). In contrast, CIH significantly increased sBP levels, but such effects were totally abolished by RD treatment (Fig. 2).

### Effects of renal denervation on CIH-induced RAS activation

**Figure 3:**
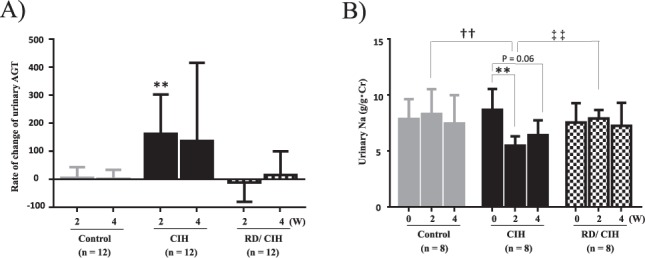
(**A**) Urinary angiotensinogen (AGT) excretion in CIH and RD/CIH: Urinary AGT levels were increased by CIH exposure, which were blocked by RD. **P < 0.01 (week 0 vs. week 2). (**B**) Urinary Na excretion in CIH and RD/CIH: Urinary Na excretion levels were diminished in the CIH model, which were returned to control levels by RD. **P < 0.01 (week 0 vs. week 2). ^††^P < 0.01 (control vs. CIH at week 2). ^‡‡^P < 0.01 (CIH vs. RD/CIH at week 2).

Since there is a strong interaction between SAS and RAS activity^4,5^, we next measured urinary excretion levels of AGT, known as a parameter of intrarenal RAS activity^16^, and assessed the effects of RD on AGT excretion. As shown in Fig. 3A, urinary AGT levels were markedly increased after CIH exposure, and were blocked by RD. Concomitant with these findings, urinary sodium excretion levels were diminished in the CIH model. RD indeed returned the sodium excretion to control levels (Fig. 3B).

### Effects of RD on CIH-induced oxidative stress

**Figure 4:**
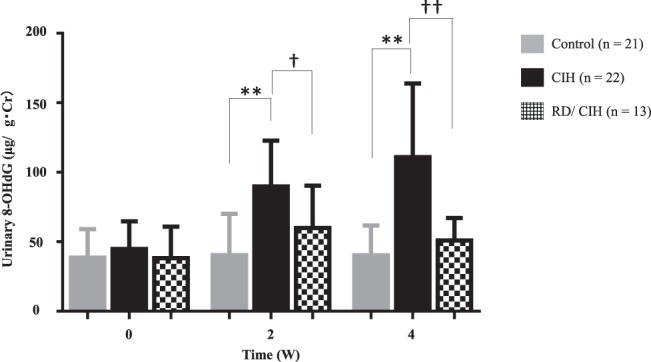
Renal oxidative stress in CIH and RD/CIH: ELISA analysis revealed that CIH mice show higher urinary 8-hydroxy-2′-deoxyguanosine (8-OHdG) excretion levels compared with control mice. RD attenuated CIH-induced urinary 8-OHdG elevation. **P < 0.01 (control vs. CIH) ^†^P < 0.05 ^††^P < 0.01 (CIH vs. RD/CIH) 8-OHdG: 8-hydroxy-2′-deoxyguanosine.

Since oxidative stress plays important roles in CIH-mediated cardiovascular injury^2^, we next assessed effects of CIH-induced renal sympathetic nerve activation on oxidative stress. As shown in Fig. 4, urinary excretion levels of 8-OHdG, known as a marker for systemic oxidative stress, were higher in the CIH model group than the Control group. RD attenuated CIH-induced urinary 8-OHdG elevation (Fig. 4).

### Effects of RD on CIH-induced endothelial dysfunction

**Figure 5:**
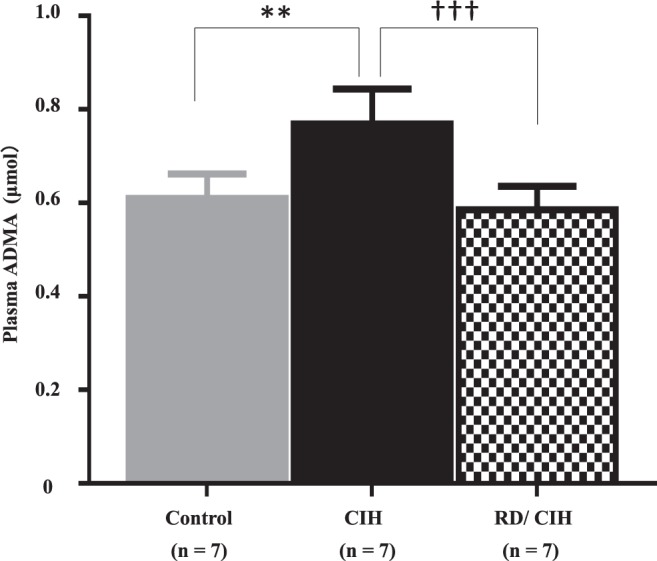
Plasma asymmetric dimethylarginine (ADMA) levels: Plasma ADMA levels at week 4 were increased by CIH exposure, and suppressed by RD treatment. **P < 0.01 (control vs. CIH). ^†††^P < 0.001 (CIH vs. RD/CIH).

To assess systemic endothelial damage, we measured plasma ADMA levels in this model. As shown in Fig. 5, plasma ADMA levels were significantly increased by CIH exposure. Such increases of ADMA were also ameliorated by RD treatment.

## Discussion

The salient findings of this study are that 1) increased urinary NE excretion was observed in the non-obese CIH model, 2) increased sBP levels were observed in the CIH model, 3) CIH exposure significantly induced RAS activation and sodium retention and increased oxidative stress and ADMA, and 4) these deleterious effects by CIH were dramatically blocked by RD treatment. These findings clearly suggest that CIH-mediated renal sympathetic nerve activation could be a major causal factor for increased oxidative stress, endothelial dysfunction and RAS activation, and therefore may contribute to the development of HT and CVD.

Consistent with a previous report^17^, sBP elevation was observed in our SAS model (Fig. 2). In addition, sBP fluctuation in the present CIH is likely associated with RAS activation and sympathetic nerve activation. We further demonstrated for the first time that RD treatment inhibited not only urinary NE elevation but also upregulation of AGT, oxidative stress, and endothelial dysfunction and subsequent BP elevation in the CIH condition. These findings strongly suggest that CIH-mediated sympathetic nerve activation could be responsible for RAS activation, increased oxidative stress, endothelial dysfunction and HT, and therefore may contribute to the development of CVD in patients with SAS. In support of our findings, the reduction of sympathetic activity in patients with SAS by treatment of Continuous Positive Airway Pressure was reported not only to ameliorate the severity of hypoxia but also to reduce sBP pressure levels in patients with SAS^18^. Moreover, Witkowski *et al*. also reported that decreases in office sBp were observed (median: −34/−13 mmHg for sBp at 6 months after the RD) in 10 patients with resistant HT and SAS. RD treatment not only exerted significant decreases in office sBP but also ameliorated the apnea-hypopnea index, Epworth Sleepiness Scale score, and plasma glucose concentration^19^. This decrease in the apnea-hypopnea index was also observed in a meta-analysis that included 49 SAS patients treated by RD^20^. In a preclinical model, Linz *et al*. showed that the increased sympathetic response associated with tracheal obstruction was attenuated with RD but not with β-blockade^21^. More recently, Kario *et al*. demonstrated the effectiveness of RD on SAS-mediated HT in a large prospective, randomized, blinded, sham-controlled trial^22^. Taken together, the activated sympathetic nerve could aggravate the severity of SAS itself and form a vicious cycle in the pathophysiology of SAS-mediated HT.

As our RD method in this study ablates both afferent and efferent renal nerves^14^, we could not clarify the independent roles of each nerve in the development of HT. Although we based our entire discussion in the present study on the assumption that RD interrupts renal sympathetic activation, some studies have indicated that signals arising within the kidney and those conveyed by renal afferents may be involved in the genesis and development of HT^23,24^. Banek CT *et al*. have recently developed a method for selective ablation of afferent renal nerves, termed renal-capsaicin (CAP), in rats, and they showed that renal-CAP markedly attenuates the development of deoxycorticosterone acetate-salt (DOCA-salt) HT. These data suggested that afferent renal nerve activity mediates the hypertensive response to DOCA-salt^23^. Therefore, further studies are needed to clarify the independent roles of each nerve in SAS-mediated HT.

ADMA is a circulating endogenous nitric oxide synthase inhibitor and well recognized as a surrogate marker for CVD^25^. In the present study, RD treatment completely blocked CIH-mediated ADMA accumulation (Fig. 5). In support of this finding, there are several reports showing that ADMA levels are elevated in patients with SAS^25,26^. In addition, a strong correlation between plasma NE and ADMA concentrations is observed in patients with end stage renal disease^27^. In patients with resistant HT, changes in sympathetic activity after RD associate with simultaneous changes in plasma levels of ADMA, strongly suggesting that the sympathetic nervous system exerts an important role in modulating circulating levels of ADMA^26^. Although we cannot clarify the underlying mechanisms how sympathetic activity could modulate ADMA levels, some SAS-related conditions such as hypoxia, RAS activation, and increased oxidative stress could explain this connection by the following evidence. Both RAS and oxidative stress are reported to diminish activity of dimethylarginine dimethylaminohydrolase (DDAH), a key limiting enzyme for ADMA degradation^28,29^, thus resulting in ADMA accumulation. It has been also reported that hypoxia itself reduced DDAH expression and subsequently increased ADMA levels in a model of pulmonary HT^30^. Accordingly, CIH-mediated tissue ischemia and activated sympathetic nerve-induced RAS and oxidative stress could be involved in DDAH dysregulation and ADMA elevation, being resulted in endothelial dysfunction in patients with SAS.

## Limitation

In the present study, although BP elevation as well as increases in surrogate markers for CVD such as AGT, 8-OHdG and ADMA was observed in our CIH model, evidence for CIH-mediated end-organ damages such as cardiac hypertrophy, atherosclerosis, renal function loss and albuminuria was missing in the observation periods (data not shown). In addition to the lack of long-term observation to evaluate the protective roles of RD against CIH-mediated organ damage, there are several limitations of this study. We realize that direct sympathetic recording is the gold standard to evaluate sympathetic nerve activity^31^. However, CIH has been shown to activate the sympathetic nerves in animal models^1,2^. In addition, studies have reported a strong relationship between urinary NE levels and intramural microelectrode recordings of muscle sympathetic nerve activity^11,32^. Therefore, we regarded NE excretion as an acceptable alternative indicator for sympathetic nerve activity in the present study. In addition, the BP measurements would be more reliable if we use telemetry. However, as described in method section, our measurements were within the CVs 5%–7% in each group, and a strong correlation between the BP levels measured by telemetry and those measured by tail cuff has been reported^33^. Therefore, we considered that the reliability of the measurement was enough in this study. In the present study, urinary AGT and Na levels were significantly increased 2 weeks after CIH exposure. Those levels after 4 weeks showed the same tendency as those after 2 weeks; however, no significant differences were observed. Although we could not clarify the mechanisms at play in the present study, certain adaptive responses for regulating AGT levels in our model may explain this finding. Lastly, we did not also evaluate the involvements of chemosensory response of the carotid body and intermittent hypercapnia, which were also known to play an important role in the development of HT in SAS^3,17^. Further studies are needed to clarify these issues.

### Perspective

In summary, CIH-mediated urinary NE elevation could be involved in increased oxidative stress, endothelial dysfunction, and RAS activation, thereby contributing to the development of HT. These results suggest that activation of sympathetic nerve system may play a major role in the development of HT and CVD, thus could be an important therapeutic target in patients with SAS.

## Materials and Methods

### Preparation of SAS mouse model

**Figure 6:**
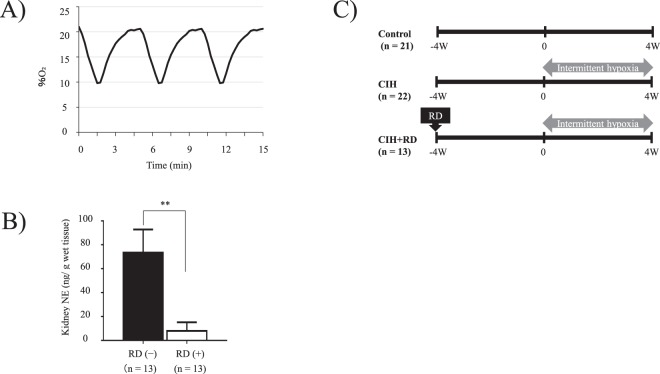
(**A**) Recorded oxygen (O_2_) profile: Recorded O_2_ profile alternating between 10% and 21% every 5 min under chronic intermittent hypoxia condition in the chamber of an *in vivo* study. (**B**) Kidney tissue NE levels after RD: RD(−) mice show significantly higher kidney tissue NE levels compared with RD(+) mice. Kidney tissue NE levels of mice subjected to RD are almost undetectable. **P < 0.01 [RD(−) + CIH vs. RD(+) +CIH]. (**C**) Protocol: We divided the mice into three groups: (1) Control in %O_2_ 21% (n = 21), (2) CIH (n = 22), and (3) RD/CIH (n = 13).

The C57BL/6 mice (CLEA, Japan, Inc.) were placed in a chamber (370 mm × 260 mm × 250 mm, 26 L, Shibata Scientific Technology Ltd, Tokyo, Japan) and exposed to CIH cycling for 8 h/day during the daytime for 4 weeks. CIH exposure was performed according to the previously described methods^34^ with minor modifications: the oxygen level was reduced from 21% to 10% over a period of 1.5 min, and returned to 21% over a period of 3.5 min. We had set this as a non-obese SAS model. The control groups were kept under normoxia and touched by human hands once a day to balance out their stress levels through direct human contact^35^. Oxygen concentration in the chamber was continuously recorded by O_2_ analyzer (XP-3180, New Cosmos Electronic Co. Osaka, Japan) (Fig. 6A).

### Renal sympathetic nerve denervation

The mice were subjected to RD under pentobarbital sodium anesthesia. Complete RD was achieved by carefully cutting and stripping all of the visible renal nerves along the renal artery and vein from the aorta to the hilum of the kidney, and painting these vessels with a solution of 10% phenol in ethanol. This method ablates the afferent and efferent renal nerves^14^. In order to minimize the impact of the invasion, the CIH was started only after 4 weeks of denervation treatment. At the end of each experiment, renal tissue was harvested and norepinephrine (NE) content was measured to confirm the completeness of RD (Fig. 6B)^15^.

### Experimental protocols of CIH

Eight-week-old male mice were used in this study. We divided the mice into three groups: (1) Control in %O_2_ 21% (n = 21), (2) CIH (n = 22), and (3) RD/CIH (n = 13) (Fig. 6C). All animal handling and experiments were performed strictly in accordance with the recommendations of the guidelines for the Care and Use of Laboratory Animals of the Juntendo University Faculty of Medicine. The experimental protocol was approved by the Animal Care and Use Committee of Juntendo University, Tokyo, Japan.

### Physiological assessments

#### Blood pressure

Systolic blood pressure (sBP) was measured in conscious, restrained mice by tail-cuff plethysmography (BP-98, Softron, Tokyo, Japan) at each time point at week 0, 2, and 4. Each recording session comprised 15–45 times of inflation and deflation per mouse. We averaged the three values with the smallest measured differences and used that value for the analysis. In addition, the first 10 cycles were acclimatization cycles, not used for analysis. Our measurements were within the CVs of 5%–7% in each group.

#### Biochemistry Measurements

For urine collection at week 0, 2, and 4, each mouse was housed overnight individually in a metabolic cage with free access to tap water (mouse metabolic cage. NATSUME SEISAKUSHO, Tokyo, Japan). At week 4, the mice were housed in metabolic cages under stabilizing conditions for the collection of 24 h urine samples, and the samples were used for urinary NE as an indirect marker of sympathetic nerve activity using high-performance liquid chromatography (Tosoh Corporation, Tokyo, Japan). Urinary creatinine concentrations were measured by immunoassay (DCA 2000 system. Bayer Diagnostics, Elkhart, Ind., USA). Urinary 8-hydroxy-2′-deoxyguanosine (8-OHdG) concentration was measured using a highly sensitive ELISA kit (JaICA, Shizuoka, Japan) and then the urinary 8-OHdG/creatinine ratio (μg/g · Cr) was calculated. Urinary angiotensinogen (AGT) concentration was measured using mouse AGT ELISA (code no.27413, IBL, Gumma, Japan), urinary AGT/creatinine ratio (μg/g · Cr) was determined, and the result was expressed as rate of change of urinary AGT. At week 4, plasma levels of ADMA were measured by ELISA kit (Immundiagnostik AG, Bensheim, Germany).

### Statistical analysis

All data were expressed as the mean ± SD. Differences between two groups were analyzed using Student’s t-test. Differences in the parameters among three groups were tested using one-way analysis of variance (ANOVA) or two-way repeated-measures-ANOVA followed by Bonferroni test. P-value of <0.05 was considered as statistically significant. Statistical analyses were performed with SPSS Version 23 (SPSS Inc., Chicago, IL, USA) and GraphPad Prism version 6 (GraphPad Inc., San Diego, CA, USA).
